# Awareness regarding the importance of calcium and vitamin D among the undergraduate pharmacy students in Bangladesh

**DOI:** 10.1186/1756-0500-6-134

**Published:** 2013-04-05

**Authors:** Riaz Uddin, Naz Hasan Huda, Yeakuty Marzan Jhanker, Tasbira Jesmeen, Mohammad Zafar Imam, Saleha Akter

**Affiliations:** 1 Department of Pharmacy, Stamford University Bangladesh, 51 Siddeswari Road, Dhaka, 1217, Bangladesh; 2 Department of Pharmacy, The University of Asia Pacific, Dhaka, Bangladesh; 3 Department of Pharmacy, Primeasia University, Dhaka, Bangladesh

**Keywords:** Calcium, Calcium inadequacy, Vitamin D, Hypovitaminosis D

## Abstract

**Background:**

Calcium and vitamin D are two important micronutrients required for maintaining proper bone health. Previous works intended to determine the status of these micronutrients in local population have reported that the people in Bangladesh are at high risk of calcium insufficiency and hypovitaminosis D related health complications. Lack of awareness and insufficient knowledge of the essentiality of these two nutrients are assumed to cause this problem in Bangladesh. The present study was designed and conducted to establish a basic understanding on the level of gap of knowledge and awareness among pharmacy students at undergraduate level in Bangladesh.

**Findings:**

A total of 713 students of Bachelor of Pharmacy course participated in the study. The students were asked about basic idea related to calcium and vitamin D and the disorders due to their deficiency, name of common foods containing calcium and vitamin D, their perception regarding the essentiality of the said nutrients etc. It was found that most of the students were familiar with the importance of calcium (98.9%) and vitamin D (99.3%) in bone health. 82.2% students know about the term osteoporosis. Unfortunately, 10.7% and 18.8% students failed to mention at least one food that is rich in calcium and vitamin D, respectively. Most of the students got familiar about the nutrients from their teachers (48.9%) and textbooks (32.8%).

**Conclusion:**

Being a student of pharmacy, the students should have more comprehensive knowledge about calcium and vitamin D. The present study indicates that the pharmacy students have lack of knowledge about calcium and vitamin D and thus it can be clearly predicted that the condition of general people may be worse.

## Findings

### Introduction

Calcium and vitamin D are two important micronutrients for maintaining proper bone health. They play a key role in preventing as well as treating different clinical conditions with excessive bone loss. They are considered to be essential for increasing peak bone mass and for minimizing age-related bone loss to reduce the risk of osteoporosis and low-trauma fractures [1]. It is evident that vitamin D is one of the 13 essential dietary vitamins and is important for intestinal absorption of calcium. Apart from its skeletal effect, vitamin D is associated with reduction of the risk of cancer, autoimmune, infectious and cardiovascular diseases [2]. Vitamin D and calcium both can be obtained directly from regular diet and supplementation. The dietary source of calcium is relatively common than that of vitamin D. Vitamin D is synthesized in our body after exposure to sunlight. But this exposure is sometimes compromised due to increased tendency to avoid sunlight exposure for cosmetic or cultural reasons and concerns about the potential risk of skin cancer [3,4].

Numerous reports have shown that relatively high proportions of people have inadequate levels of vitamin D. The extracellular health benefits of vitamin D and high prevalence of inadequate levels of vitamin D have been largely unrecognized by both physicians and patients [3]. A survey conducted by International Osteoporosis Foundation in 2007 reported that while patients are knowledgeable about the role of calcium as a bone building agent, but they are less concerned about the role of vitamin D in this process [3]. Data are available from studies on young adults [5], elderly persons; including elderly women, post-menopausal women who are at highest risk of developing osteoporosis [6-10] and healthy adolescents [11] from different countries. Some studies have also shown the prevalence of calcium and vitamin D insufficiency and the related consequences in respect to Bangladesh [12-15]. More recently, studies carried across different countries in South and Southeast Asia have found that, with few exceptions, widespread prevalence of hypovitaminosis D in both male and female and all age groups of the population [16]. Calcium deficiency rickets has been described in children from a number of countries including Bangladesh [17]. In the Indian subcontinent, rickets in infants, older children and adolescents has been reported in India, Bangladesh and Pakistan. Among the underlying causes of these diseases, maternal vitamin D deficiency and low dietary calcium were important [18]. Another two studies also reported significant prevalence of rickets in children in Bangladesh due to dietary calcium insufficiency [19,20].

There is a gap of knowledge regarding the essentiality of calcium and vitamin D among people in Bangladesh. This gap should be minimized by understanding the extent and magnitude of the problem. Bangladesh is a developing country in South Asia with a burden of large population. The people here lack the basic needs along with the need of proper healthcare. Health education system along with healthcare system is not sufficient to meet all the public health related demands in this country. A vast majority of people lack proper education, they do not know sufficient about improving their health. It is assumed that people in Bangladesh are not conscious or aware of the importance of calcium and vitamin D for proper bone health. The university going students are at the top level of their education who are believed to have some short of knowledge regarding the significance of calcium and vitamin D. The current study was designed to get an idea about the degree of shortage of knowledge regarding the use of calcium and vitamin D among the university going educated population which would help us to make an assumption about the knowledge gap exists among the general people and to take appropriate steps to fill it up.

## Methods

### Participants

All the participants in the study were undergraduate pharmacy students from different public and private universities in Dhaka and Chittagong cities of Bangladesh. The age of the participants was in the range between 18 to 20 years. The number of study population was 713 (350 were male and 363 were female). As the students came from different districts it was assumed that they correspond to a representative sample over the country. Students other than studying in Bachelor of Pharmacy were not included in the study and graduate students were not considered for the study. Other than direct questioning no data were obtained over the telephone, e-mail, mail etc. Students’ identity was also checked for authentic and valid data collection.

### Questionnaire design

A questionnaire was devised to get a gross idea about the preliminary and basic knowledge on calcium and vitamin D among the students. The questionnaire included queries about source of calcium and vitamin D rich foods, whether the subject is concerned about osteoporosis, bone mineral density (BMD) test or not, frequency of taking supplemental calcium and/or vitamin D, source of information first acquired about calcium and vitamin D. The technical terms were explained in details for better understanding of the participants. The students were informed about the purpose of the study by responsible interviewer before answering the questions. It was also made clear to the participants before question answering session that anyone did not want to participate in the study should feel free to withdraw. No multi-response answers for single-response questions were considered for data interpretation. The questionnaire was pilot tested before the survey being conducted.

### Ethical consideration

As it was a simple question based survey and no invasive methods were used, only oral consent was taken from the student. The study protocol was approved by the Institutional Ethics Committee, Stamford University Bangladesh (Reference number: SUB/SHUM/12.03).

### Statistical analysis

Statistical analysis was done using the software SPSS® version 11.5.

## Results and discussion

General knowledge about calcium and vitamin D among the students are presented in Table 1.

**Table 1:** Percentage of student’s response regarding general knowledge about calcium and vitamin D

**Topic**	**Answer (n=713)**	
	**Yes (%)**	**No (%)**
Known that calcium is an important mineral for the body	705 (98.9)	8 (1.1)
Known vitamin D is essential for normal physiological function	708 (99.3)	5 (0.7)
Familiar with the term “osteoporosis”	586 (82.2)	127 (17.8)
Being prescribed a calcium supplement ever	318 (44.6)	395 (55.4)
Being prescribed a vitamin D supplement ever	213 (29.9)	500 (70.1)
Gone for a BMI test ever	28 (3.9)	685 (96.1)

The students were asked to mention name of two foods containing calcium. 65.6% students were able to mention two food’s names which are rich in calcium, whereas 23.7% student could manage to answer 1 name and 10.7% students failed to answer the question. In case of vitamin D containing food name 43.9% students answered two names correctly, 37.3% managed to answer one name and 18.8% students failed to mention a single name of food that contains vitamin D.

The students were asked about the source of their knowledge and information they know about calcium and vitamin D. Interestingly most of the students answered that they first knew about calcium and vitamin D from either their teacher (48.9%) or from textbooks (32.8%). 9.5% answered that they first came to know about these mineral and vitamin from their family members and relatives (Table 2).

**Table 2:** Source of knowledge about calcium and vitamin D

**Source**	**% response (n=713)**
Parents, family members and relatives	9.5
Teachers	48.9
Physicians	3.4
Textbooks	32.8
Internet	2.0
Others	3.4

From the responses it was observed that both calcium and vitamin D are equally known among the male and female students. Female students are more familiar with the term osteoporosis than the male students. Only 3.1% and 1.4% of male students answered that they have been prescribed a calcium and vitamin D supplement by their physicians respectively. The percentages were 84.6% and 57.3% for calcium and vitamin D, respectively for female students. We have found no male students who have gone for a BMI test. Table 3 presents differences in responding related to male–female differences.

**Table 3:** Male female differences in the answers

**Total number of students = 713**				
	**Male = 350**		**Female = 363**	
**Question**	**Yes (%)**	**No (%)**	**Yes (%)**	**No (%)**
Known that calcium is an important mineral for the body	345 (98.6)	5 (1.4)	360 (99.2)	3 (0.8)
Known vitamin D is essential for normal physiological function	348 (99.4)	2 (0.6)	360 (99.2)	3 (0.8)
Familiar with the term “osteoporosis”	201 (57.4)	149 (42.6)	285 (78.5)	78 (21.5)
Being prescribed a calcium supplement ever	11 (3.1)	339 (96.9)	307 (84.6)	56 (15.4)
Being prescribed a vitamin D supplement ever	5 (1.4)	345 (98.6)	208 (57.3)	142 (42.7)
Gone for a BMI test ever	0 (0)	0 (0)	28 (7.7)	335 (92.3)

Vitamin D deficiency is so common as to represent a major public health problem [21]. It has re-emerged as a global public-health concern and is now presumptively linked to a range of infectious, inflammatory and neoplastic diseases throughout the life course and around the world [22]. Country specific sufficient data regarding the use, consumption of calcium and/or vitamin D for Bangladesh is not available though some studies have been conducted for the determination of vitamin D status in infants and children in different regions in Chakaria subdistrict, Cox’s Bazar, Bangladesh [19,23] and Zakiganj subdistrict in Sylhet district [14]. Calcium Research Unit, Department of Food and Environmental Sciences, University of Helsinki performed number of studies on the vitamin D status in women in Bangladesh and has observed a high prevalence of vitamin D deficiency with associated low BMD. They have recently finalized a study entitled “Bangla-D” (financed by the Academy of Finland). The research team has evaluated the vitamin D status and Bone Mineral Density (BMD) of female garments workers at Dhaka City and studied the effects of vitamin D and calcium supplementation on vitamin D and BMD status [24].

The prevalence of vitamin D insufficiency is high in South Asian countries [3,16,25]. In a recent cross-sectional study conducted by Ho-Pham and his co-workers in Vietnam, the prevalence of vitamin D insufficiency was found to be 46% in adult women and 20% in adult men [26]. They concluded that vitamin D insufficiency is common even in tropical region, and that women had a greater risk of vitamin D insufficiency than men. In Bangladesh, hypovitaminosis D is common in women regardless of age, lifestyle and clothing [27] which may explains the reason behind prescribing calcium and/or vitamin D supplements to the female students as found from the survey.

The students who are studying Bachelor of Pharmacy program are expected to have adequate knowledge of the essentiality of calcium and vitamin D and the consequences due to lack of calcium and vitamin D. From the current study it was observed that most of the students were familiar with calcium and vitamin D as food supplement. But it seems that they are only familiar to the terminologies, but they do not have sufficient knowledge about the sources from where calcium and vitamin D can be obtained. As a student of pharmacy a major portion of the students were not even familiar with the term osteoporosis.

One interesting finding of the study is that, most of the students first heard about calcium and/vitamin D from academic sources (81.7% from their teachers and textbooks). In Bangladesh the students read a chapter on the vitamins in primary education level. This reflects that the inclusion of such topics in the primary education level is justified and relevant. On the other hand it is alarming that only 9.5% of students heard the name of calcium and vitamin D from the parents, relatives or from their family. It is expected that the family will be first place for learning for a child.

## Conclusion

From this survey, we have come to know that the students who are studying pharmacy do not have adequate knowledge of essential nutrients, minerals, vitamins etc. Undergraduate pharmacy students have a long academic background related to biological science. If they have gap of knowledge about calcium and vitamin D, the general people may know little about these food supplements. The government and policy makers should pay attention about improving this situation by utilizing mass media and print media to increase awareness regarding calcium and vitamin D.

## Competing interests

The authors declare no conflict of interest.

## Authors’ contributions

RU designed the study and drafted the manuscript. NHH, MZI revised the manuscript for its critical content. MZI did statistical analysis. YMZ, TJ and SA were responsible for data acquisition and analysis. All authors read and approved the final manuscript.
